# MicroRNA let-7g acts as tumor suppressor and predictive biomarker for chemoresistance in human epithelial ovarian cancer

**DOI:** 10.1038/s41598-019-42221-x

**Published:** 2019-04-05

**Authors:** Flavia Biamonte, Gianluca Santamaria, Alessandro Sacco, Francesca Marta Perrone, Annalisa Di Cello, Anna Martina Battaglia, Alessandro Salatino, Anna Di Vito, Ilenia Aversa, Roberta Venturella, Fulvio Zullo, Francesco Costanzo

**Affiliations:** 1 Research Center of Biochemistry and Advanced Molecular Biology, Department of Experimental and Clinical Medicine, “Magna Græcia” University of Catanzaro, Campus Salvatore Venuta -Viale Europa, 88100 Catanzaro, Italy; 2 Department of Experimental and Clinical Medicine, University Magna Graecia of Catanzaro, Campus Salvatore Venuta -Viale Europa, 88100 Catanzaro, Italy; 3 Klinikum rechts der Isar, Department of Regenerative Medicine in Cardiovascular Disease, Ismaningerstr 22, Munich, Germany; 4 Unit of Obstetrics and Gynaecology, “Magna Graecia” University of Catanzaro, Catanzaro, Italy; 5 Department of Neuroscience, Reproductive Sciences and Dentistry, School of Medicine, University of Naples “Federico II”, Naples, Italy; 6 Interdepartmental Center of Services (CIS), University Magna Graecia of Catanzaro, Campus Salvatore Venuta -Viale Europa, 88100 Catanzaro, Italy

## Abstract

Remarkable deregulation of microRNAs has been demonstrated in epithelial ovarian cancer (EOC). In particular, some of the let-7 miRNA family members have been proposed as tumor suppressors. Here, we explored the functional roles of let-7g in EOC. The ectopic overexpression of let-7g in OVCAR3 and HEY-A8 EOC cells induced i) a down-regulation of c-Myc and cyclin-D2 thus promoting cell cycle arrest, ii) a reduction of Vimentin, Snail and Slug thus counteracting the progression of epithelial to mesenchymal transition, iii) a chemosensitization to *cis*-platinum treatment. Next, analysis of human EOC tissues revealed that let-7g expression was significantly reduced in tumor tissue specimens of patients with EOC compared to their non-tumor counterparts (*p* = 0.0002). Notably, low let-7g tissue levels were significantly associated with acquired chemoresistance of patients with late-stage of EOC (n = 17, *p* = 0.03194). This finding was further validated in the serum samples collected from the same cohort of patients (n = 17, *p* = 0.003). To conclude, we demonstrate that let-7g acts as tumor suppressor and might be used to disable EOC tumor progression and chemoresistance to *cis*-platinum-based chemotherapy. Furthermore, we propose that decreased expression of let-7g could serve as a tissue and serum biomarker able to predict the chemo-resistant features of EOC patients.

## Introduction

Epithelial ovarian cancer (EOC) is the leading cause of death among all gynecologic malignancies worldwide^1^. The high mortality rate of patients suffering from EOC is ascribable to various factors affecting all the different stages of the disease. Early stages of disease lack of any specific symptoms and /or early diagnostic biomarkers; thus, more than 75% of ovarian cancer patients are diagnosed at an advanced stage (International Federation of Gynecology and Obstetrics [FIGO] stage III and IV)^2,3^.At late stages, the majority of patients, although initially responding to the platinum/taxane-based chemotherapy, acquires chemoresistance^1–4^. Moreover, the protein-based clinical biomarkers, such as CA-125 and HE4, currently used to diagnose and to follow up the progression of ovarian cancer offer low specificity and sensitivity^5^. Therefore, new strategies for early detection of EOC, based on highly specific, more sensitive and non-invasive biomarkers are strongly necessary to improve EOC patients outcome. Similarly, a deeper molecular characterization of the mechanisms underlying drug resistance is essential for predicting survival and response to chemotherapeutic drugs^2,4^.

MicroRNAs (miRNAs) are short noncoding RNAs known to post-transcriptionally regulate gene expression through base-pairing to a complementary sequence of a target gene^6^. miRNAs play pivotal roles in nearly all cellular pathways orchestrating carcinogenesis, including apoptosis, cell growth and invasion, metastasis and development of chemoresistance, by acting either as oncomiR or tumor-suppressor miRNAs^7,8^. It has been widely demonstrated that the deregulation of microRNAs, both in tissue and blood, is a hallmark of each cancer cell type^8–11^. In blood, miRNAs are highly stable since they are packed in exosomes or vesicles, or bound to proteins/lipoproteins, thus representing a reliable tool to evaluate cancer development and to monitor cancer progression^8–11^.

In ovarian cancer, different miRNAs have been found aberrantly expressed^12–14^. miR-200 family members are down-regulated and play a critical role in the control of epithelial-to-mesenchymal transition (EMT); miR-34a, one of the best described p53-regulated miRNA, contributes to tumor suppression by inhibiting cellular proliferation and survival; down-regulation of let-7a, let-7e, let-7f, some of let-7 family members, is associated with aggressive behaviour of tumor and represents potential markers of invasion and metastasis in EOC^12–14^.

The let-7 miRNA family is composed of 13 members, located on 9 different chromosomes, with overlapping or distinct functions^15,16^. Since let-7 expression is reduced in almost all human cancers and this reduction is correlated with poor prognosis, they are largely described as tumor suppressors^17–25^. Let-7, indeed, negatively regulate proteins with oncogenic potential such as RAS^17,18,20^, HMGA2^20–24^, c-Myc^17,18,20^, cyclin-D2^17,18^ thus repressing cancer development, differentiation and progression. However, it has been reported that at least some of the let-7 family members may act as oncogenes. Among others, Brueckner *et al*. showed that overexpression of let-7a is associated with enhanced tumor phenotype in human cancers^26^. Aberrant expression of let-7 members has been also correlated to the acquisition of resistance to chemotherapeutic drugs^27–30^. In 2008, a discrimination of EOC patients in completely responding or non completely responding to *cis*-platinum- based chemotherapy has been proposed based on let-7i expression profile; however, the let-7i targets responsible for this effect have not been discovered^27^. More recently, Xiao M *et al*. showed that let-7e improves the response of EOC cells exposed to *cis*-platinum through the modulation of DNA double strand break repair mechanisms^29^. Therefore, the extremely variable behaviour of the different let-7 family members in tumors originating from different tissues prompts to better characterize the function of individual let-7 microRNAs in specific cancer types.

In this study, we demonstrated that let-7g acts as tumor suppressor in epithelial ovarian cancer. Indeed, ectopic overexpression of let-7g in OVCAR3 and HEY-A8 EOC cells induced cell cycle arrest, slowed down the progression of EMT and significantly improved cell response to *cis*-platinum treatment. Furthermore, we showed that let-7g amounts were significantly reduced in both tissue and plasma samples of a cohort of chemotherapy-resistant high grade serous ovarian cancer (HGSC) patients.

Overall, our results indicate that let-7g is a potential biomarker to predict chemotherapy response in ovarian cancer patients and suggest that the administration of let-7g mimics, alone or in combination with other chemotherapies, might disable tumor progression.

## Results

### Let-7g inhibits EOC cell growth through the modulation of cell cycle progression and apoptosis *in vitro*

**Figure 1:**
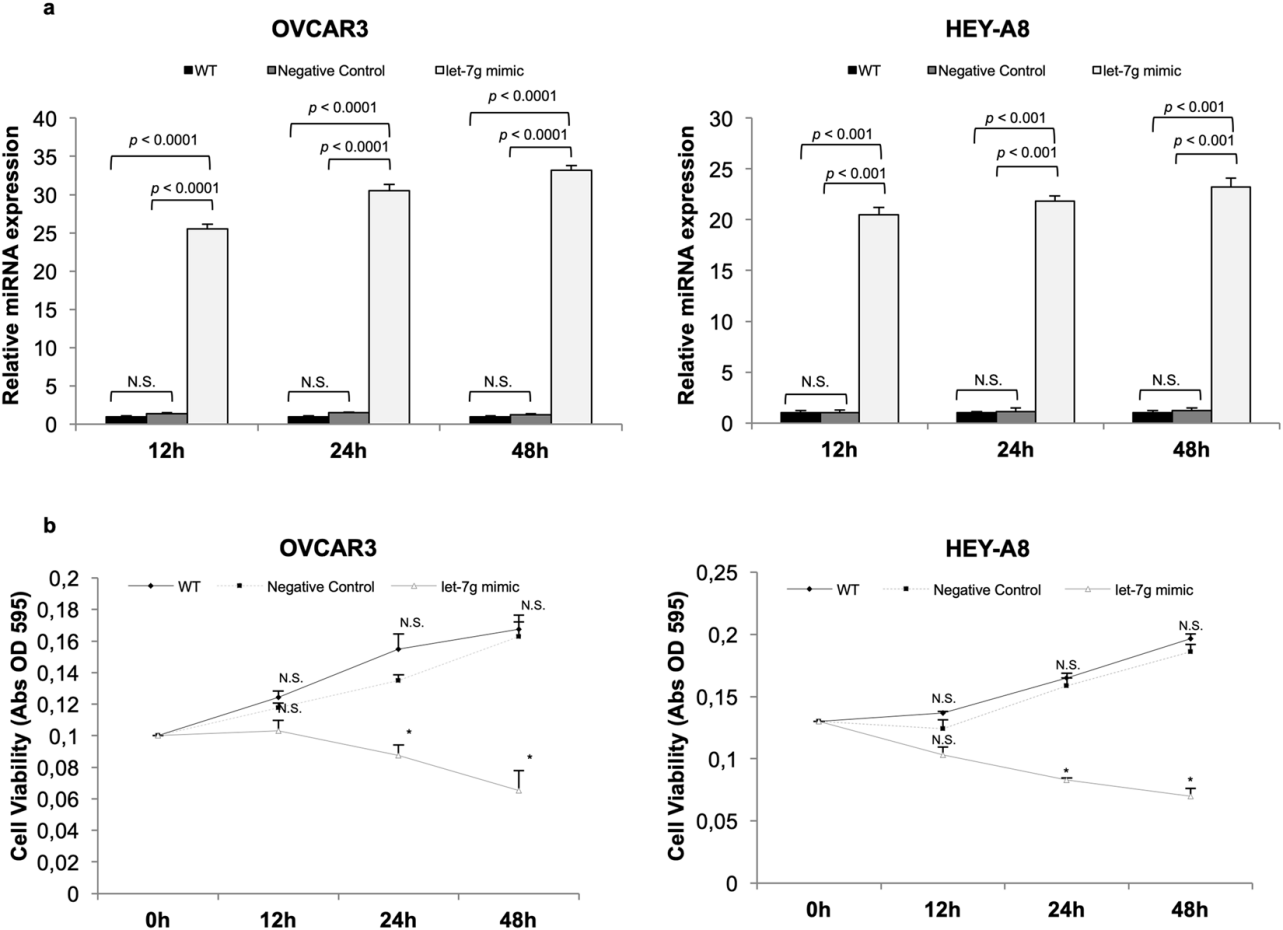
Let-7g mimic inhibits OVCAR3 and HEY-A8 cell growth. (**a)** Taqman analysis of let-7g expression in OVCAR3 and HEY-A8 cells non transfected (OVCAR3 WT and HEY-A8 WT) or transfected with either a specific let-7g mimic (OVCAR3^let-7g mimic^ and HEY-A8^let-7g mimic^) or a negative control (OVCAR3^Negative Control^ and HEY-A8^Negative Control^) at 12 h, 24 h and 48 h. N.S. Not Significant. **(b)** MTT analysis of OVCAR3 and HEY-A8 cell growth at 12 h, 24 h, 48 h upon let-7g mimic transfection. Data are shown as mean ± SD of three independent biological replicates (*p < 0.05).

**Figure 2:**
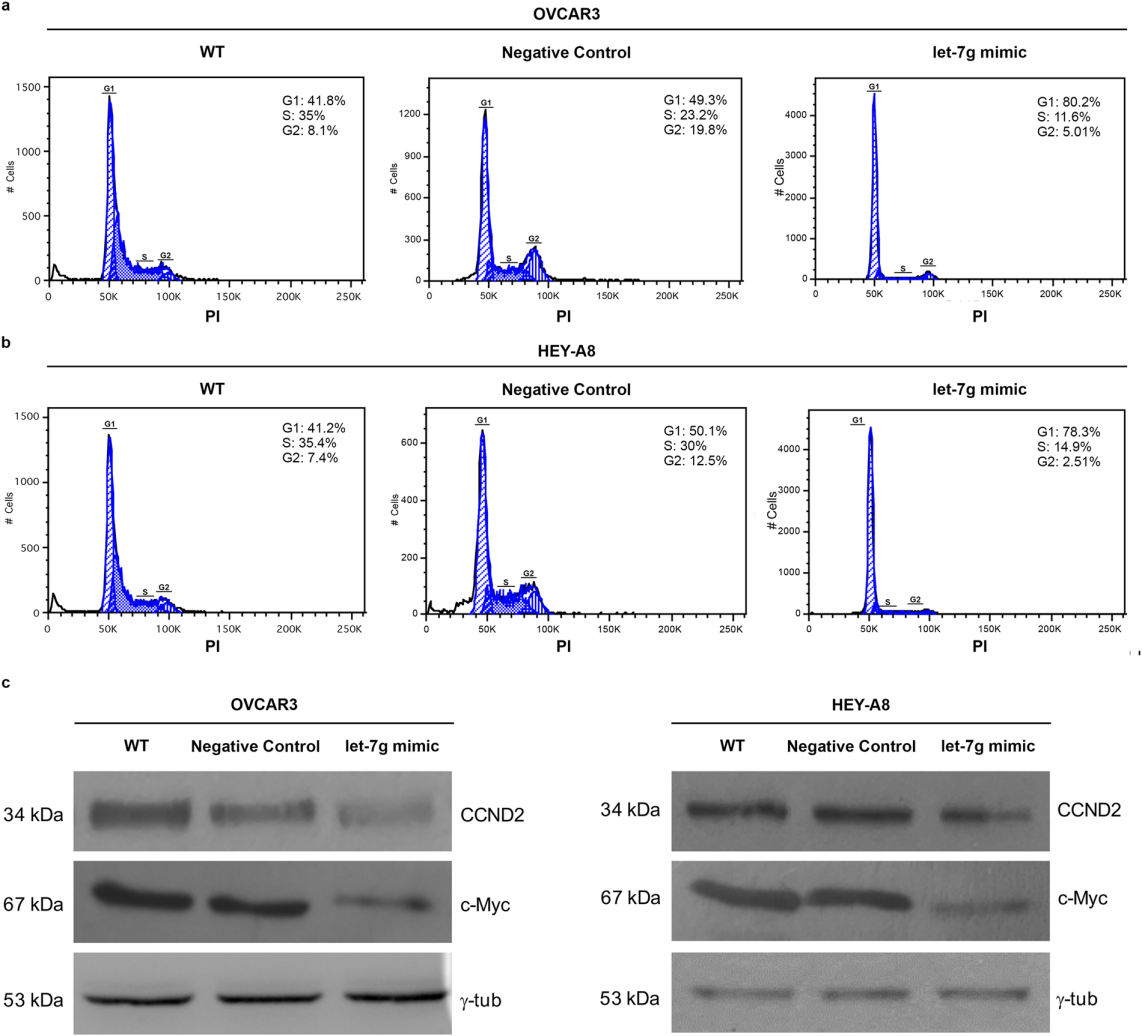
Let-7g mimic promotes cell cycle arrest in OVCAR3 and HEY-A8 cell lines. Flow cytometry analyses of OVCAR3 **(a)** and HEY-A8 **(b)** cells upon 24 h transfection with let-7g mimic and stained with PI. Each assay was performed at least three times on biological replicates. Representative plots with percentage of cells in G1, S and G2 cell cycle phases in each group is shown. **(c)** Representative western blot of cell cycle regulators c-Myc and Cyclin D2 (CCND2) in OVCAR3 and HEY-A8 control cells and OVCAR3 and HEY-A8 cells transfected with let-7g mimic for 24 h. Displayed blots are not cropped from different parts of the same gel, or from different gels.

**Figure 3:**
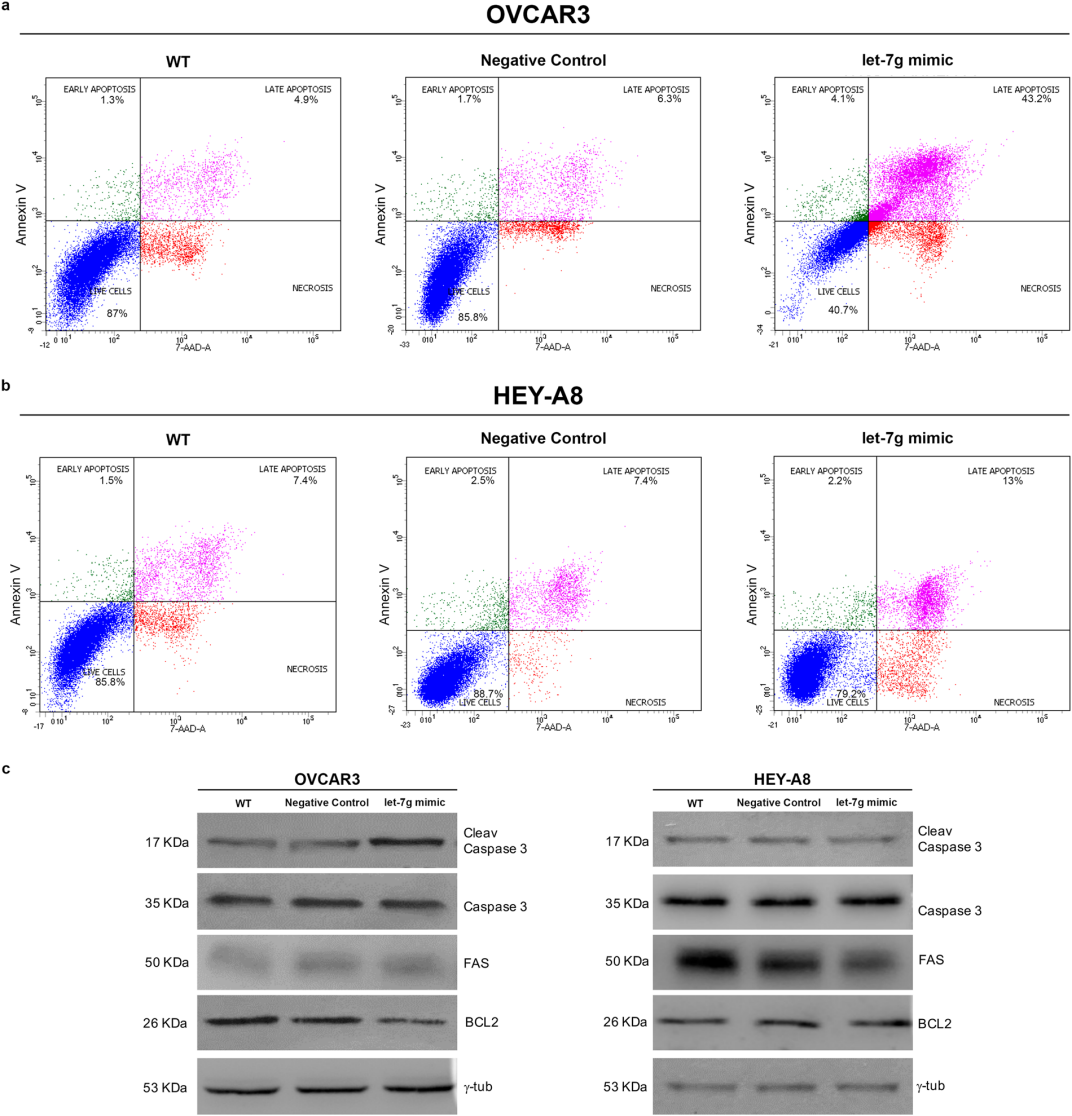
Let-7g mimic promotes apoptosis exclusively in OVCAR3 cells. Representative plots of Annexin V/ 7-AAD apoptosis assays in OVCAR3^WT^, OVCAR3^Negative Control^ and OVCAR3^let-7g mimic^ **(a)** as well as in HEY-A8^WT^, HEY-A8^Negative Control^ and HEY-A8^let-7g mimic^ **(b)** at 24 h of let-7g mimic transfection. Each assay was performed at least three times on biological replicates. (**c)** Representative western blot of apoptotic related markers Bcl-2, Caspase 3 and Fas in OVCAR3 and HEY-A8 control cells and OVCAR3 and HEY-A8 cells transfected with let-7g mimic for 24 h. Displayed blots are not cropped from different parts of the same gel, or from different gels.

We first assessed the effects of let-7g on EOC cell growth and viability. To this, OVCAR3 and HEY-A8 cells were transiently transfected with specific miR-let-7g mimic (OVCAR3^let-7g mimic^ and HEY-A8^let-7g mimic^) or its corresponding negative control (OVCAR3^Negative Control^ and HEY-A8^Negative Control^) for 12 h, 24 h and 48 h. Non-transfected OVCAR3 and HEY-A8 cells (OVCAR3 WT and HEY-A8 WT) were also used as further control. Figure 1a shows the let-7g expression levels at each transfection time point. Results from MTT analysis on three independent biological replicates show that let-7g mimic actually inhibited cell growth of both EOC cell lines, with the highest statistical significance at 24 h upon transient transfection (Fig. 1b). Next, we investigated the role of microRNA let-7g on cell cycle control and cell death. As shown by the representative FACS analysis in Fig. 2a,b, transfection of let-7g mimic for 24 h triggered a significant shift in the cell cycle distribution in both OVCAR3 and HEY-A8 cells, with an increase of the cell percentage in G1-phase (OVCAR3^let-7g mimic^ *vs* OVCAR3^Negative Control^: 79.5% ± 0.61 *vs* 49.3% ± 0.9, *p* < 0.001; OVCAR3^let-7g mimic^ *vs* OVCAR3^WT^: 79.5% ± 0.61 *vs* 42.7% ± 1.1, *p* < 0.001; HEY-A8^let-7g mimic^ *vs* HEY-A8^Negative Control^: 78.3% ± 0.34 *vs* 50.3% ± 0.67, *p* < 0.001; HEY-A8^let-7g mimic^ *vs* HEY-A8^WT^: 78.3% ± 0.34 *vs* 40.9% ± 1.67, *p* < 0.001) and a corresponding reduction of the cell percentage in S-phase (OVCAR3^let-7g mimic^ *vs* OVCAR3^Negative Control^: 12.1% ± 0.9 *vs* 23.7% ± 1.1, *p* < 0.05; OVCAR3^let-7g mimic^ *vs* OVCAR3^WT^: 12.1% ± 0.9 *vs* 33.2% ± 2.0, *p* < 0.05; HEY-A8^let-7g mimic^ *vs* HEY-A8^Negative Control^: 14.9% ± 0.9 *vs* 28.9% ± 1.7, *p* < 0.05; HEY-A8^let-7g mimic^ *vs* HEY-A8^WT^: 14.9% ± 0.9 *vs* 34.5% ± 1.5, *p* < 0.05). The analysis of two known let-7g target proteins, c-Myc and Cyclin-D2 (CCND2), highlighted their significant down-regulation in OVCAR3^let-7g mimic^ and HEY-A8^let-7g mimic^ compared to their relative control cells (Fig. 2c). The increase of let-7g levels caused a significant cell death in OVCAR3 cells, as demonstrated by the consistent increase of Annexin V^+^/7-AAD^+^ cells (OVCAR3^let-7g mimic^ *vs* OVCAR3^Negative Control^: 44.1% ± 2.7 *vs* 6.6% ± 1.8, *p* < 0.05; OVCAR3^let-7g mimic^ *vs* OVCAR3^WT^: 44.1% ± 2.7 *vs* 5.1% ± 1.2, *p* < 0.05) shown in the representative flow cytometry analysis plots in Fig. 3a. On the contrary, no pro-apoptotic effects have been observed in HEY-A8 cells (Fig. 3b). Notably, Western Blot analysis of apoptotic related markers highlighted a reduction of Bcl-2 along with an increase in cleaved Caspase 3 in OVCAR3 transfected with let-7g mimic; otherwise, let-7g overexpression was accompanied by a consistent reduction in Fas in HEY-A8 cells (Fig. 3c).

### Let-7g suppressess EMT and migration ability in OVCAR3 and HEY-A8 cells

**Figure 4:**
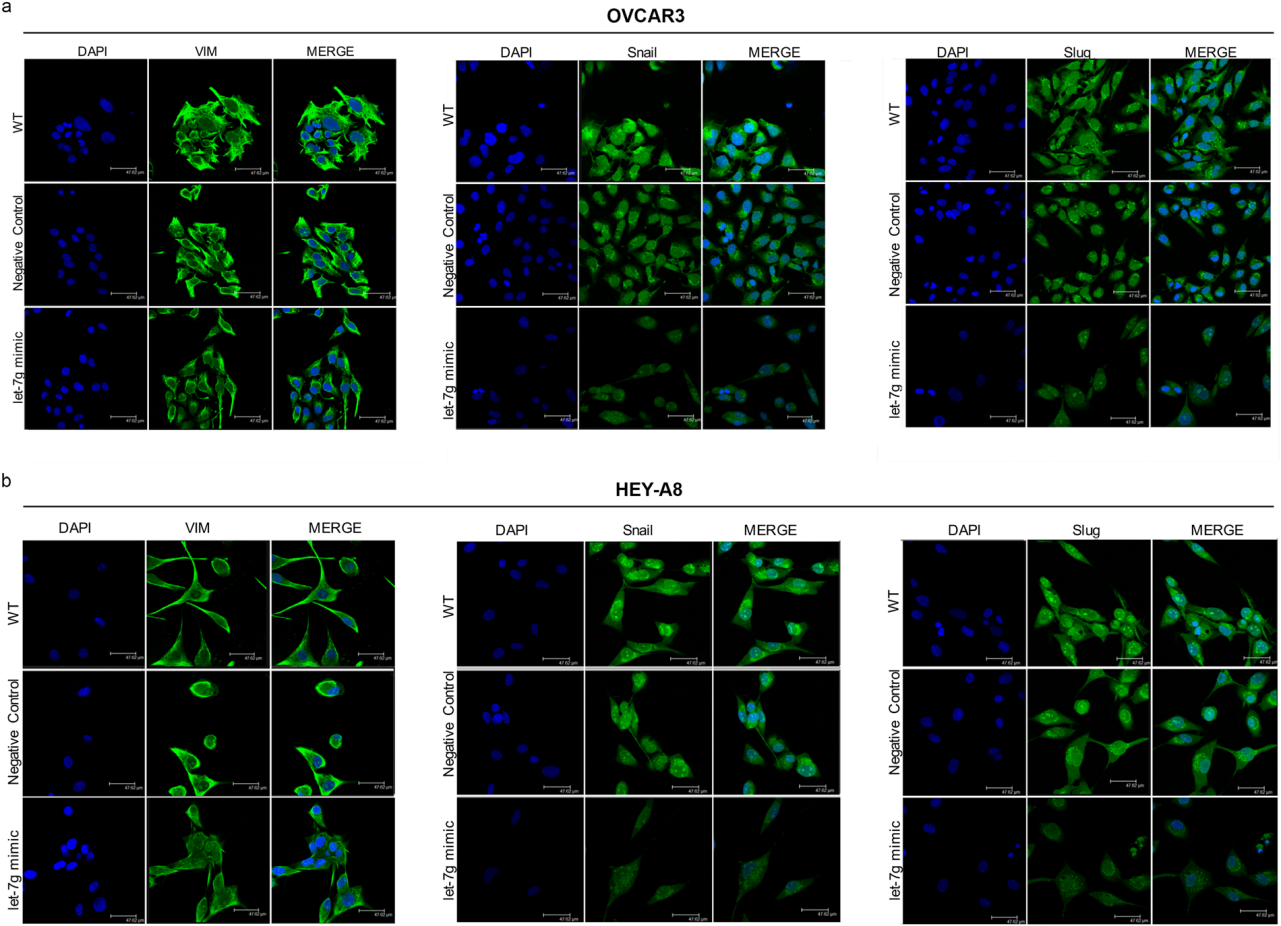
Effects of let-7g mimic on Vimentin, Snail and Slug in OVCAR3 and HEY-A8 cells measured by Immunofluorescence assays (**a)** Representative images of immunofluorescence analysis of Vimentin, Snail and Slug expression in OVCAR3 **(a)** and HEY-A8 **(b)** upon 12 h of transient transfection with let-7g mimic or miRNA mimic negative control. OVCAR3^WT^ and HEY-A8^WT^ were also analyzed as further controls. Blue, nuclei were stained with DAPI. Images were collected using Leica TCS SP2 confocal microscopy system (63x). Analyses were performed in triplicate.

**Figure 5:**
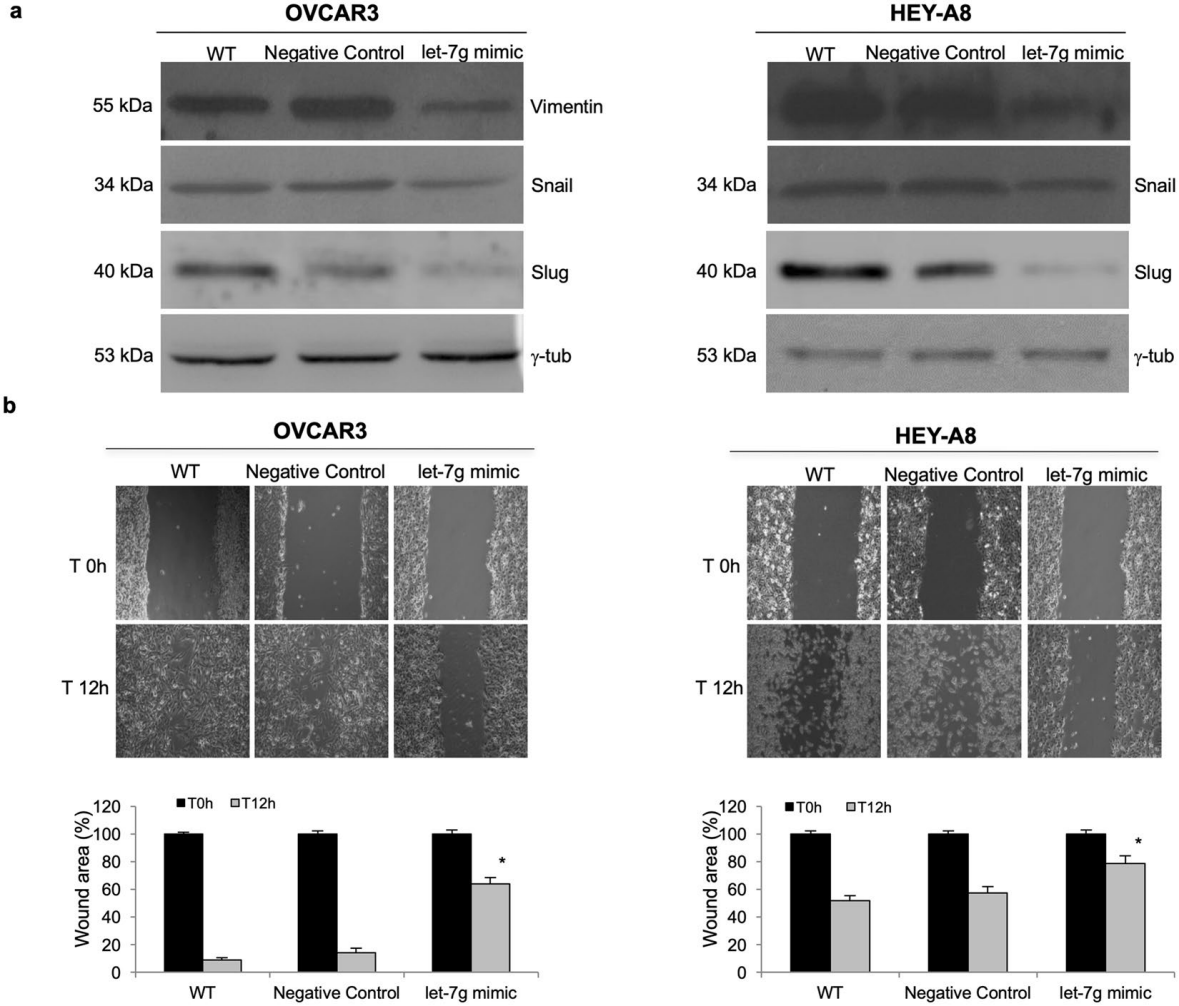
Let-7g mimic reduces Vimentin, Snail and Slug protein levels and cell motility in OVCAR3 and HEY-A8 cells. (**a**) Representative western blot analyses of the EMT-related proteins Vimentin, Snail and Slug in OVCAR3 and HEY-A8 control cells and OVCAR3 and HEY-A8 cells transfected with let-7g mimic for 12 h. Analyses were performed in triplicate. Displayed blots are not cropped from different parts of the same gel, or from different gels. (**b**) Representative images of wound healing assays in OVCAR3 and HEY-A8 control cells and OVCAR3 and HEY-A8 cells transfected with let-7g mimic. Images of cellular migration were taken at times 0 h and 12 h (magnification of 10x) using the Leica DFC420 C and Leica Application Suite Software. Analyses were performed in triplicate. Wound area quantification performed by ImageJ software is reported as %. *p < 0.05 let-7g mimic *vs* Negative Control or WT samples at 12 h.

The metastatic potential of OVCAR3 and HEY-A8 cells, in response to the overexpression of let-7g, was investigated in terms of EMT and migration ability. As shown by immunofluorescence assays in Fig. 4a,b, the transient transfection of let-7g mimic for 12 h is accompanied by the decrease of the mesenchymal marker Vimentin and the EMT-related transcription factors Snail and Slug in OVCAR3^let-7g mimic^ and HEY-A8^let-7g mimic^ compared to their relative control cells. These data were corroborated by the Western Blot analysis of the same EMT markers in Fig. 5a. The migration ability was next assessed by a triplicate set of independent wound healing assays. A representative image with relative densitometry (Fig. 5b) shows that OVCAR3^let-7g mimic^ and HEY-A8^let-7g mimic^ cells possess, at 12 h, a reduced migratory capability consistent with a slow down of epithelial to mesenchymal transition.

### Enhanced let-7g expression increases the sensitivity of EOC cells to *cis*-platinum treatment

**Figure 6:**
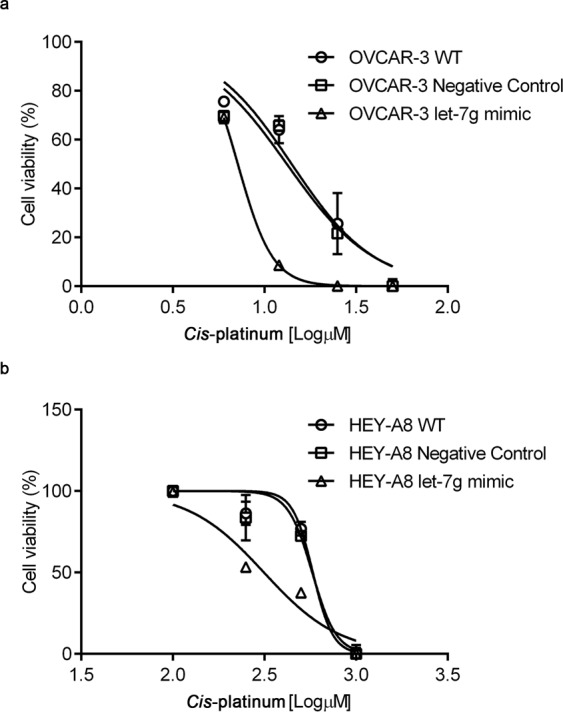
Let-7g mimic promotes *cis*-platinum sensitivity in OVCAR3 and HEY-A8 cell lines. Analysis of log EC_50_ was used to compare cytotoxicity of *cis*-platinum treatment for 24 h in OVCAR3^WT^, OVCAR3^Negative Control^ and OVCAR3^let-7g mimic^ (**a**) and in HEY-A8^WT^, HEY-A8^Negative Control^ and HEY-A8^let-7g mimic^ (**b**). Treatments were performed at least three times on independent biological replicates. *Cis*-platinum concentrations are expressed as log [µM].

Taken all together the above results strongly suggest that let-7g acts as tumor suppressor in the EOC cell lines. Thus, to explore whether this function could also affect the response to chemotherapy, OVCAR3^let-7g mimic^ and HEY-A8^let-7g mimic^ cells as well as their relative controls were exposed to serial concentrations of *cis*-platinum (OVCAR3: 6 µM, 12 µM, 25 µM, 50 µM; HEY-A8: 100 µM, 250 µM, 500 µM, 1000 µM. The overexpression of the let-7g significantly increased cell sensitivity to cis-platinum treatment in both the EOC cell lines for the majority of drug concentrations (*cis*-platinum concentration are expressed as log [µM]). Indeed, as shown in Fig. 6a, OVCAR3^let-7g mimic^ exhibited a log EC_50_ of 0.85 ± 0.00037 compared to OVCAR3^Negative Control^ which had a log EC_50_ of 1.12 ± 0.039 (*p* < 0.001) and compared to OVCAR3^WT^ which had a log EC_50_ of 1.149 ± 0.036, (*p* < 0.0001); similarly, as shown in Fig. 6b, HEY-A8^let-7g mimic^ exhibited a log EC_50_ of 2.497 ± 0.0367 compared to HEY-A8^Negative Control^ which had a log EC_50_ of 2.761 ± 0.0373 (*p* < 0.001) and compared to HEY-A8^WT^ which had a log EC_50_ of 2.764 ± 0.0466 (*p* < 0.001). EC_50_ were calculated by using GraphPad Prism® version 5.01 and analyzed by Sidak test.

### Let-7g tissue levels correlate with chemoresistance in advanced EOC patients

To further validate the tumor suppressive role of let-7g in EOC, the microRNA levels in tumor tissues obtained from 17 late-stage patients (stage III and IV) with well-documented clinical and oncological data were analyzed. At 5 years, the overall survival (OS) was 45.5% and the disease-free survival (DFS) 58.8%; the recurrence rate was 52.9%. Among the 17 patients, 9 were defined as resistant while 8 as sensitive to chemotherapy. Patient characteristics are listed in Table 1.

**Table 1:** Clinical, pathological and surgical characteristics of patients with High Grade Serous Ovarian Cancer (HGSC).

	HGSC (n = 17)
Age (years)	62.5 ± 12.0
BMI (Kg/m^2^)	33.4 ± 5.4
FIGO stage (n, %)	
*Stage IIIc*	15 (88.0)
*Stage IVa*	2 (12.0)
ASA score (n, %)	
*1*	10 (58.8)
*2*	5 (29.4)
>*2*	2 (11.8)
Primary debulking surgery (n,%)	17 (100)
Chemotherapy (n, %)	
*Platinum* + *Taxolo* + *Beva*	17 (100)
Response to chemotherapy	
*Resistant*	9 (52.9)
*Sensitive*	8 (47.1)
Major Comorbidities (n, %)	5 (29.4)
Follow-up (months)	29.7 ± 21.2

**Figure 7:**
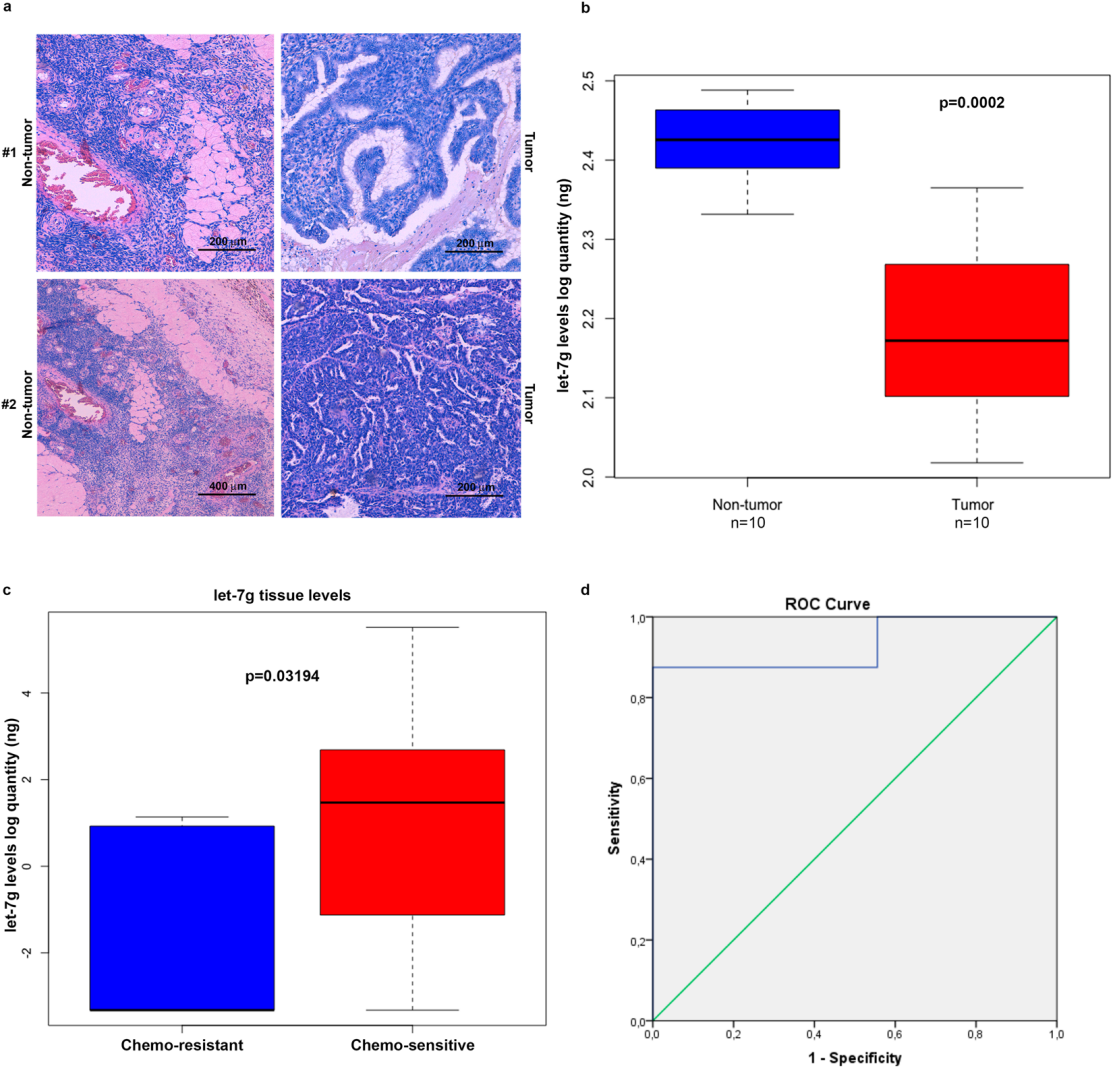
Let-7g is down-regulated in tumor tissues and discriminates chemoresistant from chemosensitive HGSC patients. **(a)** Representative images of ovarian cancer tissues and relative non-tumor counterparts. **(b)** Box Plot depicting let-7g levels, expressed as log quantity (ng), as assessed by absolute TaqMan analysis in tumor tissues (n = 10) and their relative non-tumor tissues (n = 10) when available (*p* = 0.0002). **(c)** TaqMan analysis of let-7g tissue levels, expressed as log quantity (ng), in chemo-resistant HGSC patients (n = 9) and chemo-sensitive HGSC patients (n = 8). Data were analyzed by Kruskal–Wallis test and represented as box plot. (*p* = 0.03194). **(d)** ROC curve analysis for let-7g to discriminate HGSC patients who respond to chemotherapy from those who not-completely respond to chemotherapy.

First, absolute TaqMan analysis of let-7g expression levels was performed in EOC tumor tissues (n = 10) and matched adjacent non-tumor tissues when available (n = 10) (see representative immunoistochemical images in Fig. 7a). As shown in the box plot in Fig. 5b, statistical Kruskal–Wallis test highlighted that let-7g was significantly down-regulated in tumor tissues compared to their non-tumor counterparts (*p* = 0.0002). We also observed that, within the tumor tissue specimens, let-7g amount was significantly lower in the patients with resistance to chemotherapy compared to chemo-sensitive ones (*p* = 0.03194)(Fig. 7c). Receiver operating characteristics (ROC) curve analysis was performed to evaluate the accuracy of tissue let-7g to distinguish chemoresistant EOC patients from those who were sensitive to chemotherapy. An area under the curve (AUC) of 0.931 had a high discriminatory power (95% CI: 0.792–1.000, p < 0.05) (Fig. 7d).

### Low levels of serum let-7g is associated with chemoresistance in patients with advanced EOC

**Figure 8:**
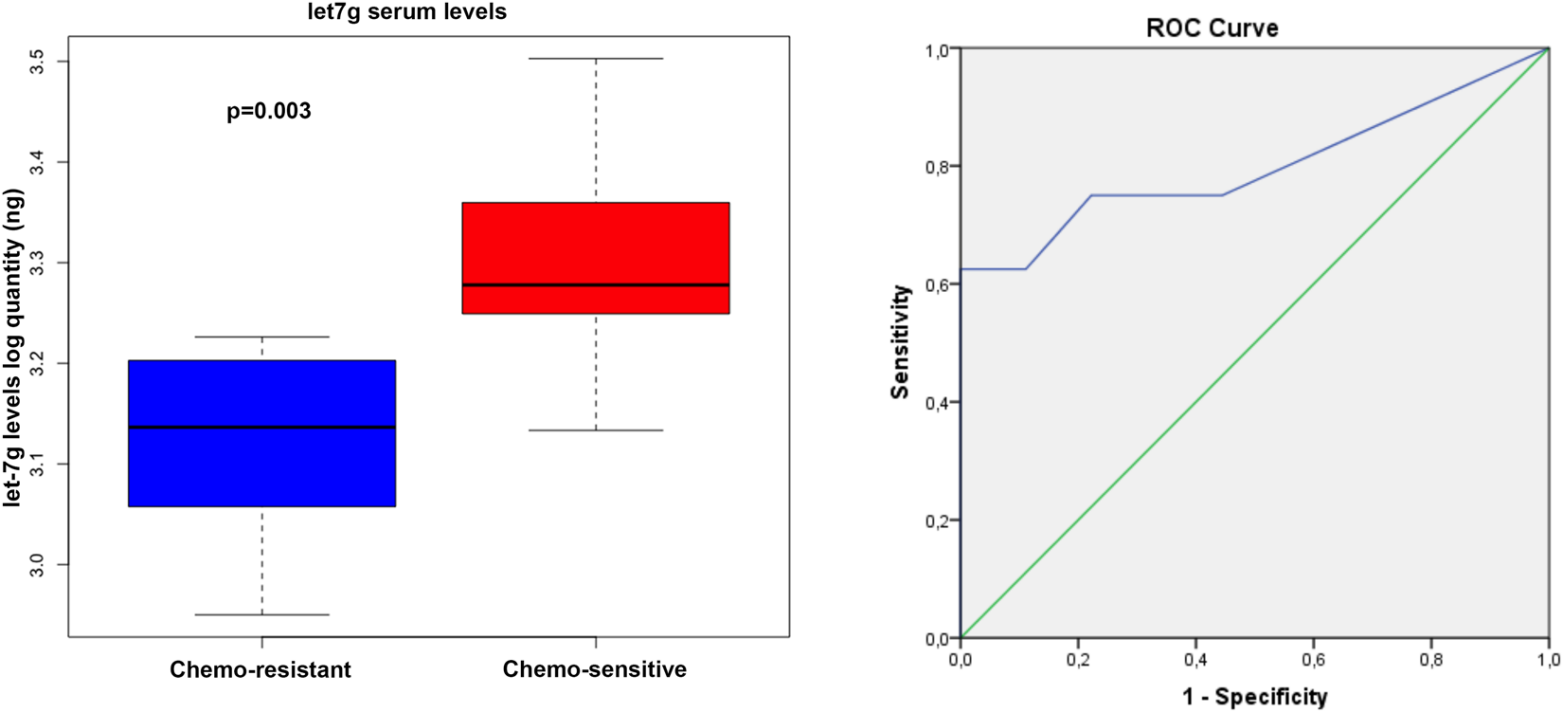
Let-7g serum levels discriminate chemoresistant from chemosensitive HGSC patients. TaqMan analysis of let-7g serum levels, expressed as log quantity (ng), in chemo-resistant HGSC patients (n = 9) and chemo-sensitive HGSC patients (n = 8) (left). Data were analyzed by Kruskal–Wallis test and represented as box plot (*p* = 0.003). ROC curve analysis for let-7g to discriminate HGSC patients who respond to chemotherapy from those who not-completely respond to chemotherapy (right).

Driven by the above results, we further examined let-7g expression levels in serum samples collected from the same cohort of patients at the time of diagnosis. The correlation analysis between the level of serum let-7g and the response to chemotherapy highlighted that let-7g serum amount was significantly lower in patients with resistance to chemotherapy compared to chemo-sensitive ones (*p* = 0.003) (left in Fig. 8). ROC curve analysis showed that AUC of 0.799 had a high discriminatory power (95% CI: 0.563–1.000, p < 0.05) (right in Fig. 8).

## Discussion

Ovarian cancer usually presents in advanced stages with a bad prognosis and a high mortality rate^1–4^. Primary debulking surgery has been the standard of care in this disease for a long period. More recently, the administration of a platinum/taxol-based neoadjuvant chemotherapy followed by surgical cytoreduction has proven to be a more promising treatment strategy for the management of advanced EOC patients^31^. However, ovarian cancer is one of the most referred cancer types developing drug resistance that, therefore, dramatically reduces the neoadjuvant chemotherapy benefits^1–4^. The identification of reliable biomarkers enabling clinicians to distinguish the chemoresistant from chemosensitive subjects, among stage III and IV EOC patients, is one of the main goals of translational medicine.

In the recent years, the enormous efforts in the molecular characterization of EOC pathogenesis have led to the identification of a subset of miRNAs which might represent novel strategies for early detection, diagnosis, and treatment of this disease^12–14^, although for most of them the prospective validation is still pending.

MicroRNAs Let-7 family, that insist on multiple cellular pathways mainly connected with neoplastic transformation, are frequently found down-regulated or even lost in many cancers, hence the definition of tumor suppressor miRNAs^15–17^. Let-7 microRNAs belong to a family composed by 14 highly homologous members, whose expression is often co-regulated. However, it has been demonstrated that the relationship between let-7 family and neoplastic transformation is a complex phenomenon in which individual members may either show a different expression level within the same cancer cell either play distinct roles in different types of malignancies^15–17^.

In this work, we investigated the functional role of a single member of let-7 family, let-7g, on epithelial ovarian cancer. Taken all together experimental assays performed on OVCAR3 and HEY-A8 EOC cell lines identify microRNA let-7g as a tumor suppressor able to consistently disable many of the cancer hallmarks. First, we found that let-7g overexpression induced a significant reduction of cancer cell growth. This effect was associated with a partial arrest in G_0_/G_1_ cell cycle phase in both OVCAR3 and HEY-A8 cells. It has been widely reported that let-7 microRNA family universally interferes with cell growth through directly targeting several oncogenic proteins involved in cell cycle progression including c-Myc, CDC25A, CDK6 and cyclin D2^16,17,32^. Consistent with these data, we found that the overexpression of let-7g remarkably down-regulated c-Myc and cyclin-D2 in OVCAR3 and HEY-A8 cells. The role of let-7 family in the regulation of programmed cell death appears, instead, context-dependent due to its ability to act on different target genes. Geng, L *et al*. showed that in human colorectal cancer cells let-7 inhibits apoptosis by decreasing the expression of Fas^33^ while Zhang, H *et al*. demonstrated, in CRC cell lines, that let-7 promotes apoptosis by targeting some members of anti-apoptotic Bcl-2 protein family^34^. In our experimental model, let-7g overexpression differently affects apoptosis in the two cell lines: in OVCAR3 cells, the miRNA promoted an increase in the apoptotic rate due to its preferential activity on Bcl-2 while in the apoptotic resistant HEY-A8 cells let-7g mimic preferentially targeted FAS, decreasing its amount. These data provide further confirmation of the context-specific activity of let-7 also in ovarian cancer cell lines.

Next, we demonstrated that in EOC cell lines let-7g overexpression cut back the epithelial to mesenchymal transition (EMT) process and reduced the cell motility. These activities were accompanied by a break-down of the mesenchymal marker Vimentin and by a diminished expression of the two EMT-related transcription factors Snail and Slug. The identification of let-7g as suppressor of EMT in ovarian cancer raises some interesting questions. To the best of our knowledge none of the three proteins has been described as direct target of let-7 family. Hence, we performed target prediction analyses by PicTar, miRTar and TargetScan bioinformatic prediction softwares that showed the lack of any regions of complementarity between Vimentin, Snail and Slug mRNAs and let-7g (data not shown), thus suggesting the existence of indirect regulatory mechanisms. Interestingly, let-7g overexpression in OVCAR3 and HEY-A8 cells was accompanied by the up-regulation of other miRNAs involved in EMT control and ovarian cancer pathogenesis, namely let-7b, -e and -i and miR-200b (see Supplementary Fig. S1)^35,36^. The dissection of the metabolic routes connecting let-7g with this repertoire of EMT-related miRNAs require further and deeper future studies.

The tumor suppressive effects of let-7g in EOC cell lines is further confirmed by the enhanced sensitization of both OVCAR3 and HEY-A8 cells exposed to *cis*-platinum treatment. In ovarian cancer, c-Myc has been reported as central hub in the *cis*-platinum resistance, so that its specific targeting has been proposed as possible strategy to overcome chemoresistance^37^. The diminished expression of c-Myc upon let-7g mimic transfection in both cell types might constitute one of the molecular ground of this phenomenon.

Our *in vitro* data, indicating the tumor suppressive role of let-7g in EOC, were supported by the analysis of let-7g expression levels *ex vivo*. Recently, Yang N *et al*. have reported let-7i as an important miRNA involved in the chemotherapy response of EOC patients, thus proposing it as putative prognostic biomarker^27^. Our data support and expand these results; in fact, although the small cohort size of 17 EOC patients, we show that another member of let-7 family, let-7g, is able to discriminate, both in tissue specimens and serum samples, patients with risk to develop resistance from those who completely respond to platinum-taxane chemotherapy.

Overall, this study demonstrates that let-7g acts as tumor suppressor by reducing epithelial ovarian cancer cell aggressiveness. The *in vitro* analyses add Vimentin, Snail and Slug to the repertoire of the molecules involved in let-7g downstream networks. Furthermore, the here presented *ex vivo* analyses, albeit needing to be confirmed in larger patient cohorts in future studies, suggest that tissue let-7g detection and potentially the quantification of its circulating levels, might be useful to guide decision making on usage of neo-adjuvant chemotherapy in patients with advanced epithelial ovarian cancer.

## Methods

### Cell lines and cell culture

Epithelial ovarian cancer cell lines OVCAR3 and HEY-A8 were obtained from the American Type Culture Collection (ATCC, Rockville, MD, USA). OVCAR3 cells were maintained in RPMI 1640 media (Sigma-Aldrich, St. Louis, MO, USA) while HEY-A8 were maintained in DMEM media (Sigma-Aldrich, St. Louis, MO, USA). Both culture media were supplemented with 10% (v/v) fetal bovine serum (FBS) (Thermo Fisher Scientific, Waltham, Massachusetts, USA) and 1% (v/v) penicillin and streptomycin (Sigma-Aldrich, St. Louis, MO, USA), in adherent cultures at 37 °C in a humidified 5% CO_2_ atmosphere. Each cell line has been routinely examined for *Mycoplasma* contamination.

### Patients and specimens

We selected a group of 17 patients with High Grade Serous Ovarian Cancer (HGSC) who were treated at the Unit of Gynaecologic Oncology, Magna Graecia University, Germaneto, and Pugliese-Ciaccio Hospital, Catanzaro, Italy, between April 2013 and March 2016. Data were retrieved from the charts, collected and tabulated. Tissue and serum samples of patients were retrieved from our bio-bank to perform analysis of miRNA let-7g expression.

Procedures carried out in this study were in accordance with the guidelines of the Helsinki Declaration on human experimentation and good clinical practice (CGP). Approval by the “Pugliese-Ciaccio” institutional review board (IRB number: AOPC12404) was obtained before starting patient’s enrollment. Furthermore, an informed consent was obtained from all patients before processing their data from the time of hospitalization, even if data did not include any personal identifying information. Inclusion criteria were as follow: availability of clinical data and biological samples; stage IIIc-IV HGSC surgically staged. Patients with previous or concurrent cancer located in other sites, known genetic susceptibility to gynecologic or non-gynecologic cancers (BRCA1-2 carriers, associated polyposis conditions (APC), Fanconi syndrome) or positive family anamnesis for ovarian and/or breast cancer were excluded. Biological samples consist in surgical tissue and serum aliquots. Specifically, tumoral and healthy surgical samples consist in tissues fixed in 4% paraformaldehyde and subsequently embedded in paraffin.

### Transient transfection of let-7g mimic

Hsa-let-7g-5p *mir*Vana® miRNA mimic (MC11758) and negative control were purchased from Thermo Fisher Scientific (Waltham, Massachusetts, USA). OVCAR3 and HEY-A8 were seeded in a 24-well or 6-well plate to reach a 50% to 70% confluence the next day. After 24 h, cells were transfected with 50 nM of hsa-let-7g-5p mimic (OVCAR3^let-7g mimic^ and HEY-A8^let-7g mimic^) or mirVana mimic negative control (OVCAR3^Negative Control^ and HEY-A8^Negative Control^) using Lipofectamine 2000 transfection reagents (Thermo Fisher Scientific, Waltham, Massachusetts, USA) following the manufacturer’s instructions. Cells were incubated in the medium containing the transfection mixture up to 48 h. Non transfected cells (OVCAR3^WT^ and HEY-A8^WT^) were also used as further controls. Experiments were performed at least three times.

### RNA isolation and qRT-PCR analysis

Total RNA isolation and single-stranded complementary DNA (cDNA) generation were performed as previously reported in Biamonte *et al*.^35^. Quantification of miRNA let-7g was achieved by TaqMan MicroRNA assay kit (Thermo Fisher Scientific, Waltham, Massachusetts, USA) with specific primer sets for U6 snRNA (Assay ID: 001973), hsa-let-7g-5p (Assay ID: 002282), hsa-miR-200b-5p (Assay ID: 002251), hsa-let-7i-5p (Assay ID: 002221), hsa-let-7b-5p (Assay ID: 002619), hsa-let-7e-5p (Assay ID: 002406) (Thermo Fisher Scientific, Waltham, Massachusetts, USA) according to the manufacturer’s instructions. U6 snRNA was used as internal normalizer.

Absolute TaqMan Analysis was also used to determine the expression of let-7g in the 17 tumor tissue specimens and serum samples. Briefly, starting from a sample of known template concentration, a 5-point 10 fold serial standard curve was prepared, and the concentration of all other samples were calculated by simple interpolation of each threshold cycle (Ct) into this standard curve. MiRNA expression data are reported as log quantity (ng) and represent the mean of three independent technical replicates.

### Western blotting

Total cell lysates were prepared using RIPA buffer^38,39^. Each protein sample (40–60 μg) was separated by 10–15% SDS–PAGE and then transferred to nitrocellulose membranes. Membranes were incubated with primary antibodies at 4 °C overnight. Primary antibodies against c-Myc (C33, sc-42), Cyclin D2 (B-6, sc-376676), Vimentin (V9, sc-6260), SNAI1 (E-10, sc-393172), SLUG (A-7, sc-166476) and Bcl-2 (sc-509) were purchased from Santa Cruz Biotechnology (Santa Cruz Biotechnology, Dallas, Texas (Santa Cruz Biotechnology, Dallas, Texas) while antibodies against Fas (4233 S) and Caspase 3 (9662 S) were purchased from Cell Signalling Technology, Leiden, Netherlands). Membranes were then washed and incubated, for 2 h, with secondary antibodies HRP-conjugated goat anti-mouse IgG (sc-2005) and HRP-conjugated goat anti-rabbit IgG (sc-2357) (Santa Cruz Biotechnology, Dallas, Texas), and immunoreactive bands were visualized with the ECL Western blotting detection system (Santa Cruz Biotechnology, Dallas, Texas). To ensure equal loading of proteins was used a goat polyclonal anti-γ-Tubulin antibody (C-20) (1:3000; sc-7396, Santa Cruz Biotechnology). Experiments were performed three times and representative images are reported.

### *Cis*-platinum treatment

OVCAR3 WT, OVCAR3^Negative Control^, OVCAR3^let-7g mimic^, HEY-A8 WT, HEY-A8 ^Negative Control^ and HEY-A8^let-7g mimic^ were seeded in a 24-well plate in antibiotic-free medium. *Cis*-platinum or mock PBS alone were added into the medium at various concentrations (OVCAR3: 6 µM, 12 µM, 25 µM, 50 µM; HEY-A8: 100 µM, 250 µM, 500 µM, 1000 µM). The cell viability analysis, using MTT assay, was performed 24 h post-drug addition. Treatments were performed at least three times on independent biological replicates. EC50 was calculated by using GraphPad Prism® version 5.01.

### MTT assay

For the MTT assays, 3-[4,5-Dimethylthiaoly]-2,5-diphenyltetrazolium bromide (MTT) (Sigma Aldrich, St. Louis, MO, USA) was used. Briefly, OVCAR3 and HEY-A8 cells (25 × 10^3^ cells/well) were seeded into 24-well plate. After 24 h starvation, both cell lines were transfected with let-7g mimic (OVCAR3^let-7g mimic^ and HEY-A8^let-7g mimic)^ or mirVana mimic negative control (OVCAR3^Negative control^ and HEY-A8^Negative control)^. OVCAR3 WT and HEY-A8 WT cells (i.e. non-tranfected cells) were also used as further controls. Briefly, fresh MTT 2 mg/mL (Sigma Aldrich, St. Louis, MO, USA), re-suspended in PBS, was added to each well containing OVCAR3 and HEY-A8 at 12 h, 24 h and 48 h upon transient transfection with let-7g mimic or negative control; fresh MTT 2 mg/mL (Sigma Aldrich, St. Louis, MO, USA), re-suspended in PBS, was also added to each well containing OVCAR3 WT and HEY-A8 WT. After 2 h incubation, culture medium was discarded and replaced with 200 μL of isopropanol. Optical density was measured at 595 nm in a spectrophotometer. For each sample, MTT assay was performed in triplicate.

### Cell Cycle analysis

A total 2 × 10^6^ cells were fixed with 100% ethanol and stored at 4 °C for overnight. Cells were rehydrated with PBS for 10 min at RT and then cells were stained with propidium iodide (PI) staining solution contained with 50 μg/ml PI (Sigma Aldrich, St. Louis, MO, USA), 100 μg/ml DNase-free RNase A (Calbiochem, La Jolla, CA), and 0.01% NP-40 (USB, Cleveland, OH) in PBS for 60 min at room temperature. Stained cells were analyzed for cell cycle analysis in BD LSRFortessa^TM^X-20 (BD Biosciences, San Jose, CA) and FlowJo software.

### Apoptosis analysis

For identifying the cells actively undergoing apoptosis a double staining with Annexin-V and 7-AAD was performed using Alexa Fluor®488 Annexin V/ Dead Cell Apoptosis Kit (Thermo Fisher Scientific, Waltham, Massachusetts, USA) according to the manufacturer’s instructions. After staining, cells were incubated at room temperature for 15 min in the dark. Each tube was diluted with 400 µl of Annexin Binding Buffer and then cells were analyzed by flow-cytometry using the BD LSRFortessa^TM^X-20 (BD Biosciences, San Jose, CA) and FACSDiva 7.0 program (BD Biosciences, San Jose, CA).

### Immunofluorescence

OVCAR3 and HEY-A8 cells were cultured on cover slip and then transfected with let-7g mimic or negative control. Upon 24 h, cells were collected and immunofluoresce assay was performed as previously reported by Aversa I *et al*.^40^. Primary antobodies Vimentin (V9, sc-6260), SNAI1 (E-10, sc-393172), SLUG (A-7, sc-166476) (Santa Cruz Biotechnology, Dallas, Texas) was incubated for 2 h. The slides were mounted on microscope slides using a mounting solution ProLong Gold antifade reagent (Thermo Fisher Scientific). Images were collected using a Leica DM-IRB/TC-SP2 confocal microscopy system (63x).

### Wound healing assay

OVCAR3 and HEY-A8 cells were seeded in a 6-well plates and transfected with let-7g mimic or negative control. OVCAR3^WT^ and HEY-A8^WT^ cells were used as further controls. After 6 h, a (yellow) pipette tip was used to make a scratch, for simulating a wound. At 0 h and 12 h cells were monitored and images of wound healing were captured (magnification of 10x) using the Leica DFC420 C and Leica Application Suite Software. Migration rates were quantified using ImageJ.

### Statistical analysis

Results are expressed as mean ± SD, and analyzed using the unpaired Student’s t-test. Let-7g levels in tumor *vs* adjacent non tumor tissue specimens, as well as let-7g tissue and serum levels in chemo-resistant *vs* chemo-sensitive HGSC patients were compared using the non-parametric Kruskal–Wallis test to identify statistical differences between groups. *p* ≤ 0.05 was considered to be significant. Statistical Package for Social Sciences Version 14.0.1 (SPSS Inc., Chicago, IL, USA) was clinical correlation analyses. GraphPad Prism® version 5.01 was used to calculate EC50; Sidak test was used to identify statistical significance in the EC50 values. The receiver operating characteristic (ROC) was generated for let-7g using both tissues and serum samples. The area under the curve (AUC) values and 99% confidence intervals (CIs) were calculated to determine the specificity and sensitivity. ROC Curve analysis was performed by using SPSS statistics 20.

## Supplementary information

Supplementary Figure 1

## Data Availability

All data generated or analysed during this study are included in this published article.
