# Supplementary material for: MicroRNA let-7g acts as tumor suppressor and predictive biomarker for chemoresistance in human epithelial ovarian cancer
Source: Sci Rep. 2019 Apr 5;9:5668. doi: 10.1038/s41598-019-42221-x (PMC6450929; doi:10.1038/s41598-019-42221-x)

MicroRNA let-7g acts as tumor suppressor and predictive biomarker for chemoresistance in human epithelial ovarian cancer

Flavia Biamonte*[1,2], Gianluca Santamaria[3], Alessandro Sacco[2], Francesca Marta Perrone[2], Annalisa Di Cello[4], Anna Martina Battaglia[2], Alessandro Salatino[2], Anna Di Vito[2], Ilenia Aversa[2], Roberta Venturella[4], Fulvio Zullo[5], Francesco Costanzo[2,6]

Supplementary Figure 1. Taqman analysis of let-7b, let-7e, let-7i and miR-200b microRNAs in OVCAR3 and HEY-A8 cells transfected with let-7g mimic and their relative controls. Taqman analysis of microRNAs let-7b, -e, -I and miR-200b in OVCAR3[WT], OVCAR3[Negative Control], OVCAR3l[et-7g mimic], HEY-A8 [WT], HEY-A8 [Negative Control] and HEY-A8 [let-7g mimic] The experiments were performed at least three times in biological replicates. Data are reported as mean ±SD. * $p<0.05$ let-7g mimic *vs* Negative Control samples; N.S. Not Significant Negative Controls *vs* WT samples.

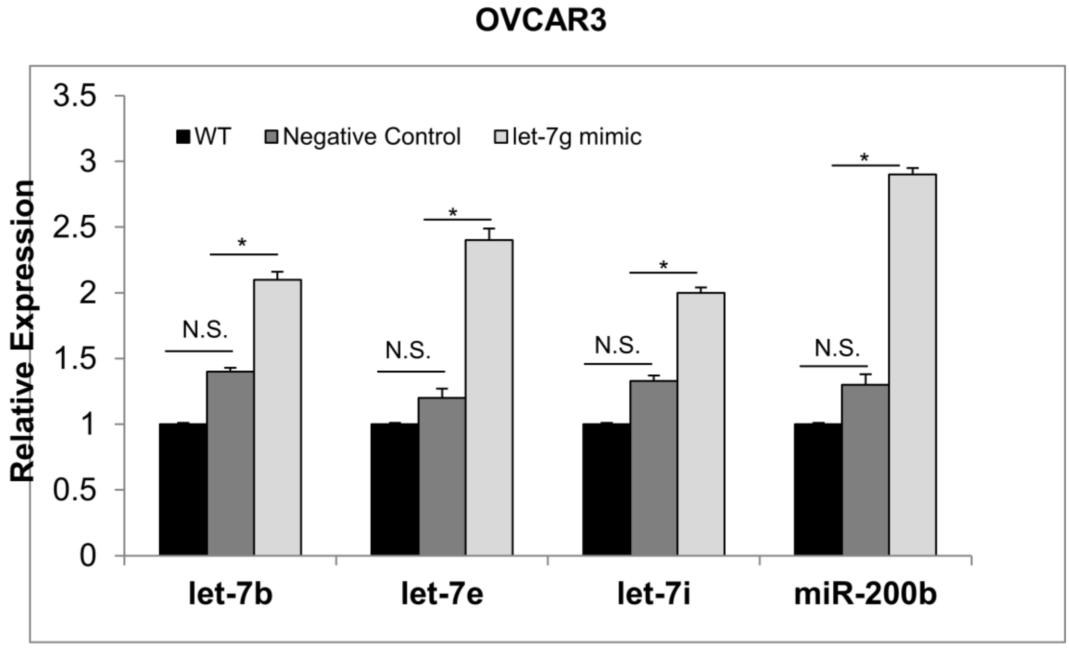

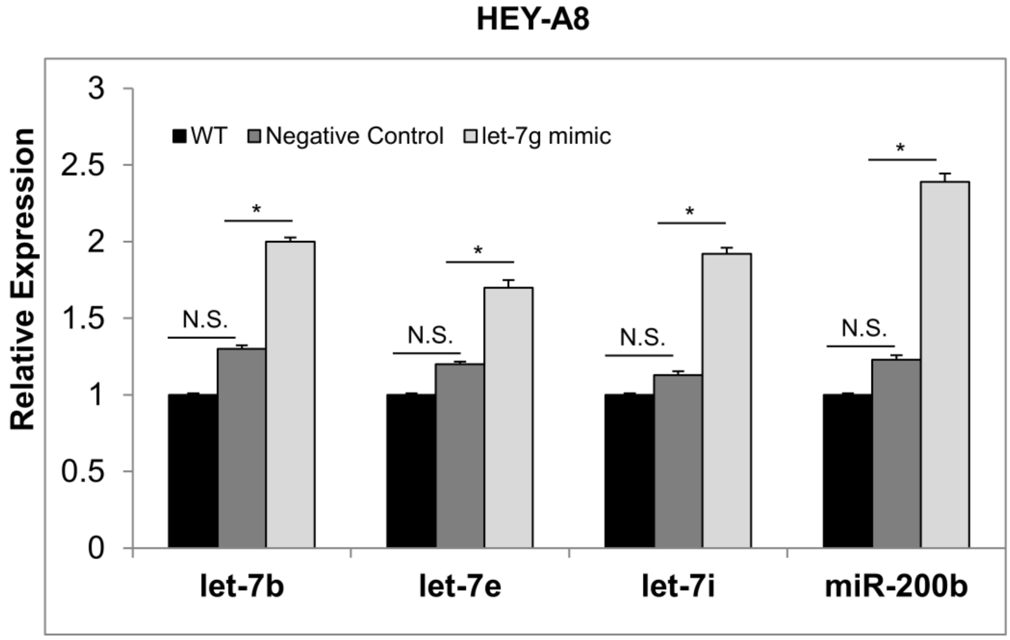

Supplement: Supplementary file 1 — Supplementary Figure 1 [file 41598_2019_42221_MOESM1_ESM.pdf]
